# GADD45A Does Not Promote DNA Demethylation

**DOI:** 10.1371/journal.pgen.1000013

**Published:** 2008-03-07

**Authors:** Seung-Gi Jin, Cai Guo, Gerd P. Pfeifer

**Affiliations:** Division of Biology, Beckman Research Institute of the City of Hope, Duarte, California, United States of America; The Babraham Institute, United Kingdom

## Abstract

Although DNA methylation patterns in somatic cells are thought to be relatively stable, they undergo dramatic changes during embryonic development, gametogenesis, and during malignant transformation. The enzymology of DNA methyltransferases is well understood, but the mechanism that removes methylated cytosines from DNA (active DNA demethylation) has remained enigmatic. Recently, a role of the growth arrest and DNA damage inducible protein GADD45A in DNA demethylation has been reported [1]. We have investigated the function of GADD45A in DNA demethylation in more detail using gene reactivation and DNA methylation assays. Contrary to the previous report, we were unable to substantiate a functional role of GADD45A in DNA demethylation. The mechanism of active DNA demethylation in mammalian cells remains unknown.

## Author Summary

During mammalian development, genome-wide DNA demethylation occurs both in developing germ cells and in fertilized oocytes. This rapid DNA demethylation is an active process that occurs in the absence of DNA replication. The mechanism of active DNA demethylation represents a conundrum for researchers in this field, i.e. the breakage of a carbon-carbon bond to remove a methyl group from the DNA cytosine ring appears energetically unfavorable, and the elimination of approximately 30 million 5-methylcytosine bases from both DNA strands within a short time window raises questions about the maintenance of genome stability during this process. Recently, it has been reported that the protein GADD45A, a small acidic protein that has been implicated in the DNA damage response, plays a crucial role in promoting active DNA demethylation in several mammalian cell lines. We noticed that GADD45A does not fulfill one likely requirement for a mammalian DNA demethylase factor in that it is not expressed in oocytes or zygotes. We then investigated the role of GADD45A in DNA demethylation using methylated reporter plasmids and DNA methylation analysis of several endogenous genes in cell lines overexpressing GADD45A. Contrary to the previous report, we were not able to demonstrate a role of GADD45A in DNA demethylation. The activity that promotes DNA demethylation at a genome-wide level in mammals remains to be identified.

## Introduction

Mammalian DNA methylation patterns are thought to be generally quite stable throughout cell divisions due to faithful maintenance of DNA methylation patterns during DNA replication [2]. There is, however, published data showing that in somatic cells methylated CpGs can be converted to unmethylated CpGs in the absence of DNA replication [3],[4],[5],[6],[7]. This replication-independent DNA demethylation would imply the existence of a mammalian DNA demethylase enzyme that can either actively remove the methyl group from 5-methylcytosine or can remove the entire methylated base or nucleotide, perhaps in a base excision repair-like pathway. Direct breakage of a carbon-carbon bond seems energetically unfavorable. If not strand-specifically coordinated, the excision repair pathway would put the genome at risk for DNA double strand breakage. The mechanistic steps and proteins involved in DNA demethylation in mammals are currently unknown. In plants, a demethylase pathway involving a DNA glycosylase activity has been identified [8],[9],[10] but these proteins do not appear to have mammalian homologues. The mammalian cytidine deaminases AID and APOBEC1 in vitro have 5meC deaminase activity [11] and the deaminated base, thymine, may be removed by base excision repair pathways.

One of the best pieces of evidence for an active DNA demethylation pathway in mammalian cells comes from studies of pre-implantation mouse embryos. It has been shown that the paternal genome becomes almost completely demethylated within less than six hours after fertilization. This process must be independent of DNA replication. Astonishingly, the maternal genome resists this genome-wide demethylation process [12],[13],[14],[15].

The nature of the mammalian DNA demethylase has remained obscure. Earlier, the recombinant MBD2b protein has been reported to possess DNA demethylase activity in vitro [16], although this finding has so far not been repeated in other laboratories [17],[18]. Staining of 5-methylcytosine with an antibody in fertilized oocytes containing an *Mbd2* knockout allele gave results that were the same as wild type controls [19] arguing that MBD2 is not involved in zygotic DNA demethylation.

Recently, it was reported that the GADD45A protein promotes demethylation of CpG-methylated DNA [1]. *GADD45A* (Growth arrest and DNA-damage-inducible gene 45 alpha) is a gene induced by a variety of growth arrest conditions and DNA damaging agents. The GADD45A protein is an 18 kDa acidic nuclear protein involved in maintenance of genomic stability, DNA repair, cell cycle checkpoints and suppression of cell growth [20],[21]. Barreto et al reported that GADD45A has a key role in active DNA demethylation. GADD45A overexpression activated a methylation-silenced reporter plasmid and promoted DNA demethylation [1].

Here, we have further investigated the role of GADD45A in DNA demethylation. However, we were not able to substantiate a role of GADD45A in demethylation of mammalian DNA.

## Results

### Gene expression profile analysis in mouse developmental stages using the UniGene database

The best evidence for active DNA demethylation occurring in mammalian genomes comes from studies of fertilized oocytes where it has been shown that the paternal genome is actively demethylated before the onset of DNA replication [12],[13]. Barreto et al identified GADD45A as a putative factor for DNA demethylation by screening a *Xenopus* cDNA expression library for cDNAs able to activate a methylation-silenced luciferase reporter gene [1]. However, in contrast to mice, the paternal genome of *Xenopus* is not subjected to active demethylation of 5-methylcytosine immediately after fertilization [22].

We hypothesized that *Gadd45a* should be expressed in the oocyte or zygote if it plays a role in the mammalian DNA demethylation pathway. The gene expression profile of *Gadd45a* in mouse developmental stages was assessed by examining the UniGene database. Although this database cannot replace direct analysis of gene expression, it can provide a useful guide. Also, while it is possible that a demethylase pathway is exclusively expressed in oocytes/zygotes, this may not necessarily be the case, as tissue-specific posttranscriptional factors may modify protein levels or activity. With this caveat noted, we observed that *Gadd45a* is expressed in late developmental stages or in somatic tissues (Table 1). We compared the expression profile of *Gadd45a* with the expression profile of *PGC7*/*Stella*, a protein known to be important for the prevention of demethylation of the maternal genome in fertilized oocytes [23]. As expected, *PGC7*/*Stella* was expressed at high levels in oocytes, unfertilized ova, zygotes and in preimplantation embryos but *PGC7*/*Stella* mRNA was virtually absent at later embryonic stages and in adult tissue (Table 1). Thus, the expression of *Gadd45a* and *PGC7*/*Stella* are almost mutually exclusive, a situation not expected if *Gadd45a* functions in mammalian demethylation, at least in zygotes.

**Table 1:** The expression profile of *GADD45A* and *PGC7/Stella* in mouse developmental stages^a^

Developmental Stage	Gadd45a (Mm.389750^b^)		*PGC7/Stella* (Mm.27982)	
	TPM^c^	Gene EST/Total EST	TPM	Gene EST/Total EST
oocyte	0	0/19546	869	17/19546
unfertilized ovum	0	0/20165	99	2/20165
zygote	0	0/28822	1179	34/28822
pre-implantation embryo	28	4/142770	413	59/142770
post implantation embryo	0	0/43031	0	0/43031
mid-gestation embryo	17	3/175666	0	0/175666
late gestation embryo	23	2/86464	0	0/86464
fetus	21	13/607548	1	1/607548
neonate	18	2/105302	9	1/105302
juvenile	3	1/290147	0	0/290147
adult	11	12/1060463	0	0/1060463

a) Expression profiles were determined by analysis of EST counts from the Mus musculus UniGene database as of August 2007. b) UniGene ID, c) TPM indicates transripts per million.

### A role for GADD45A in activation of methylation-silenced reporter plasmids?

Over the past decade, it has become a difficult issue to explore the biological function of GADD45A [21]. Its role in the induction of apoptosis is unclear. We have not seen apoptotic cells by TUNEL staining in cells transfected with GADD45A and have not seen a substantial cell cycle arrest by FACS analysis of cells transfected with GADD45A (data not shown). The phenotype of cells with deleted Gadd45a is centrosome amplification and mitotic failure [20]. GADD45A interacts with the mitotic kinase Aurora A [24]. The transfected GADD45A protein does indeed interact with Aurora A (Figure S1) attesting to the functionality of the protein.

**Figure 1:**
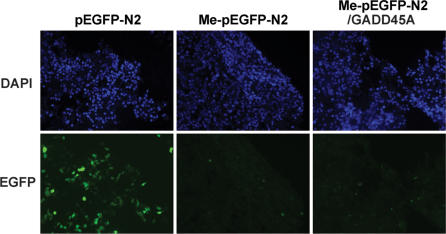
GADD45A does not reactivate a methylation-silenced EGFP reporter gene. HEK293 cells were transiently transfected with an unmethylated (pEGFP-N2) or CpG-methylated (Me-pEGFP-N2) plasmid and the GADD45A expression vector. Fluorescence microscopy indicates that expression of GADD45A does not promote reactivation of EGFP.

Barreto et al have shown that overexpression of GADD45A can lead to reactivation of a methylation-silenced EGFP reporter gene [1]. We first tested if overexpression of GADD45A has an effect on the expression of methylated reporter plasmids in somatic cells. As a simple assay for detecting demethylation activity, in vitro methylated promoter and reporter genes have been used to detect methylation changes in mammalian cell lines [25],[26]. As done by Barreto et al. [1], we used this assay system to quantify a change in the state of expression of the methylated *EGFP* reporter plasmid (pEGFP-N2) when GADD45A was overexpressed in HEK293 cells. The EGFP reporter gene, which is controlled by the CMV promoter, was in vitro methylated at all CpG sites with SssI DNA methylase and transiently transfected or co-transfected with a *GADD45A* mammalian expression vector into HEK293 cells. After 48 hours, expression of EGFP was determined by fluorescence microscopy (Figure 1). The methylated reporter gene was not transfected at a lower efficiency than the unmethylated one as assessed with a co-transfected luciferase expression plasmid (data not shown). While EGFP was expressed from the unmethylated plasmid, the methylated EGFP plasmid was not expressed. EGFP expression in *GADD45A* overexpressing HEK293 cells was observed at a level similar to that of the control, suggesting that *GADD45A* does not have an effect on the methylation-silenced EGFP reporter plasmid and does not release methylation silencing (Figure 1).

### Direct analysis of GADD45A-induced DNA demethylation with in vitro methylated plasmids

**Figure 2 pgen-1000013-g002:**
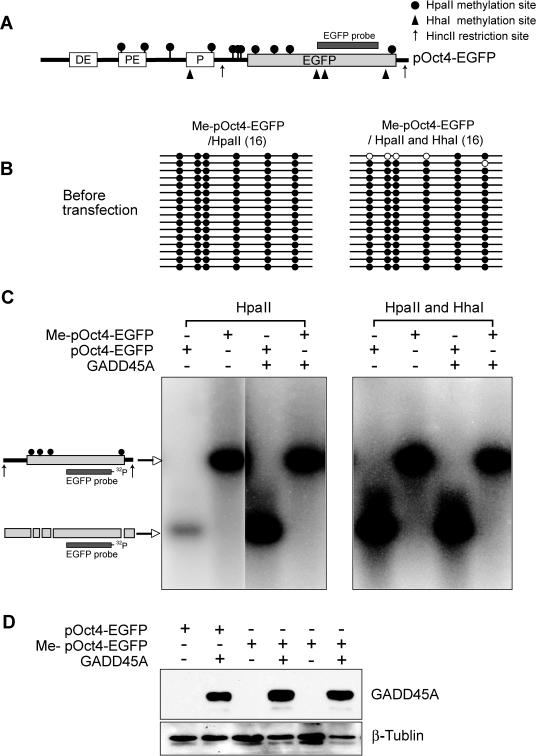
Overexpression of GADD45A does not promote demethylation of a methylated pOct4-EGFP plasmid. A. Schematic diagram of the pOct4-EGFP plasmid (not to scale). The 2.4 kb Oct4 promoter sequences are upstream of the EGFP gene. DE is the distal element, PE is the proximal element, and P is the minimal promoter of Oct4. This plasmid was methylated in vitro with HpaII or HpaII + HhaI. Black circles indicate HpaII sites and black triangles indicate HhaI sites. B. Confirmation of the methylation status of the six HpaII sites (black circles in panel A) by sodium bisulfite sequencing. Closed circles are methylated sites and open circles are unmethylated sites. C. Southern blot assay of plasmids recovered from HEK293 cells after transient transfection of the methylated and unmethylated pOct4-EGFP plasmid with and without GADD45A overexpression. The plasmids were digested with HincII and HpaII after in vitro methylation of the plasmid with HpaII (left panel) or HpaII and HhaI (right panel) prior to transfection. Digestion of the recovered methylated plasmid with HpaII does not lead to cleavage indicating that demethylation did not occur in HEK293 cells that overexpress GADD45A. D. Confirmation of GADD45A expression in transfected HEK293 cells by Western blotting with anti-GADD45A antibody. Beta-tubulin was probed as a loading control.

**Figure 3 pgen-1000013-g003:**
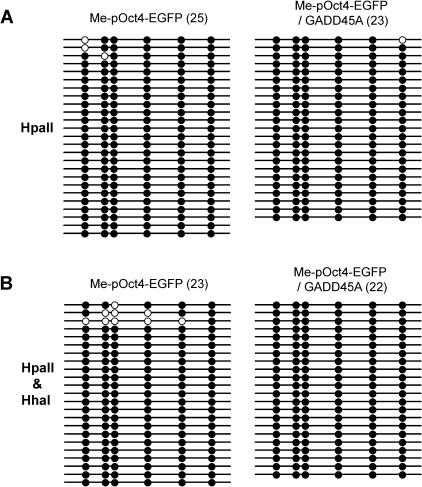
Bisulfite sequencing of the methylated pOct4-GFP plasmid recovered from HEK293 cells transfected in the absence or presence of the GADD45A expression vector. A. The plasmid was methylated with HpaII methylase only. B. The plasmid was methylated with HpaII and HhaI methylases. The recovered DNA was treated with sodium bisulfite and the pOct4-EGFP target sequences containing the six HpaII sites (see Figure 2A) were amplified from bisulfite-treated DNA. The PCR products were cloned and between 22 and 25 individual molecules were sequenced. Closed circles mark methylated HpaII sites and open circles mark unmethylated HpaII sites. Overexpression of GADD45A (right panels) did not induce demethylation of the plasmid.

It was reported that GADD45A promotes active DNA demethylation in mammalian cell lines [1]. We replaced the CMV promoter in the pEGFP-N2 plasmid with the 2.4-kb mouse Oct4 gene promoter (Figure 2A). DNA demethylation has been previously studied with murine Oct4-EGFP gene constructs [27],[28],[29]. We asked whether GADD45A overexpression induces demethylation of the in vitro methylated mouse Oct4 promoter plasmid in HEK293 cells. The methylation status of the Oct4 promoter-EGFP reporter gene was determined by Southern blot analysis with methylation-sensitive restriction enzyme digestion, as was done by Barreto et al. [1] (Figure 2), and by bisulfite sequencing (Figure 3). The pOct4-EGFP construct was in vitro methylated with HpaII and HhaI methylases. The efficiency of methylation of the Oct4-EGFP plasmid was tested by bisulfite sequencing and it was judged to be at least 95% (Figure 2B). In vitro methylated pOct4-EGFP plasmids were recovered from control and GADD45A overexpressing HEK293 cells 48 hours after transfection with or without *GADD45A* mammalian expression vector. The GADD45A overexpression in HEK293 cells was verified by Western blot analysis with GADD45A-specific antibody (Figure 2D). The transiently transfected HEK293 cells were grown under conditions of serum starvation to separate active DNA demethylation from passive demethylation events due to any DNA replication. It is noted that Barreto et al observed demethylation in both dividing and non-proliferating cells [1]. The recovered plasmids were digested with the methylation-sensitive restriction enzyme HpaII, which cleaves the sequence 5′-CCGG only when it is not methylated. The digested fragments were subjected to Southern blot analysis with a ^32^P-labeled EGFP probe to determine the degree of demethylation as shown in Figure 2C. However, we failed to detect HpaII-digested fragments from methylated plasmids recovered from GADD45A overexpressing cells. This demonstrates that no significant demethylation occurred in methylated and transfeced pOct4-EGFP plasmids in HEK293 cells with or without GADD45A overexpression. This assay was repeated with HpaII-only methylated pOct4-EGFP, because Barreto et al. showed HpaII-digested EGFP gene fragments when HpaII-only methylated plasmid was recovered from GADD45A overexpressing cells [1]. But, as shown in Figure 2C, we also failed to detect demethylation of pOct4-EGFP when it was in vitro methylated with HpaII only. This result was further confirmed by a more sensitive technique, sodium bisulfite sequencing. As shown in Figure 3, bisulfite sequencing of six HpaII methylation sites within and upstream of the EGFP gene clearly shows that the level of methylation on the pOct4-EGFP plasmid, which was in vitro methylated by either HpaII single methylation or by HpaII and HhaI double methylation, was not affected by expression of GADD45A. These data suggest that GADD45A does not promote active DNA demethylation.

### Effect of GADD45A on the endogenous mouse Oct4 promoter

**Figure 4 pgen-1000013-g004:**
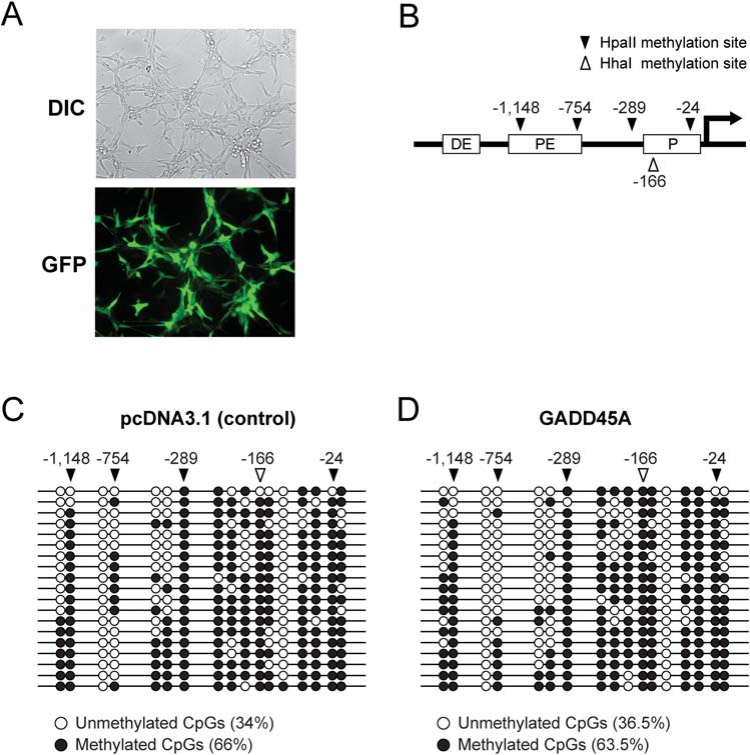
GADD45A does not promote demethylation of the endogenous pOct4 promoter in NIH3T3 cells. A. The transfection efficiency of NIH3T3 cells was analyzed with an EGFP expression vector and was calculated to be ∼60%. DIC, differential interference contrast image; GFP, green fluorescence. B. Schematic diagram of the mouse Oct4 promoter region. DE is the distal element, PE is the proximal element, and P is the minimal promoter of Oct4. Black triangles indicate HpaII sites and the open triangle shows a HhaI site. C and D. Bisulfite sequence analysis of all CpGs in the Oct4 upstream region. Closed circles are methylated CpGs and open circles are unmethylated CpGs. C, control vector transfected cells; D, GADD45A expression vector transfected cells.

It was reported that GADD45A promotes substantial demethylation of the endogenous Oct4 promoter in mouse NIH3T3 cells although the transfection efficiency for these cells was only ∼30% [1]. Using Amaxa nucleofection technology, we achieved a transfection efficieny of over 50% for NIH3T3 cells as tested with a GFP expression plasmid (Figure 4A). However, despite of the higher transfection efficiency, we were not able to see any significant demethylation of the Oct4 promoter using bisulfite sequencing analysis (Figure 4). In control vector transfected cells, 34% of the CpGs were unmethylated and in GADD45A-transfected cells, 36.5% of the CpGs were unmethylated, a difference which was statistically not significant.

### Does GADD45A induce DNA demethylation at other genomic loci?

**Figure 5 pgen-1000013-g005:**
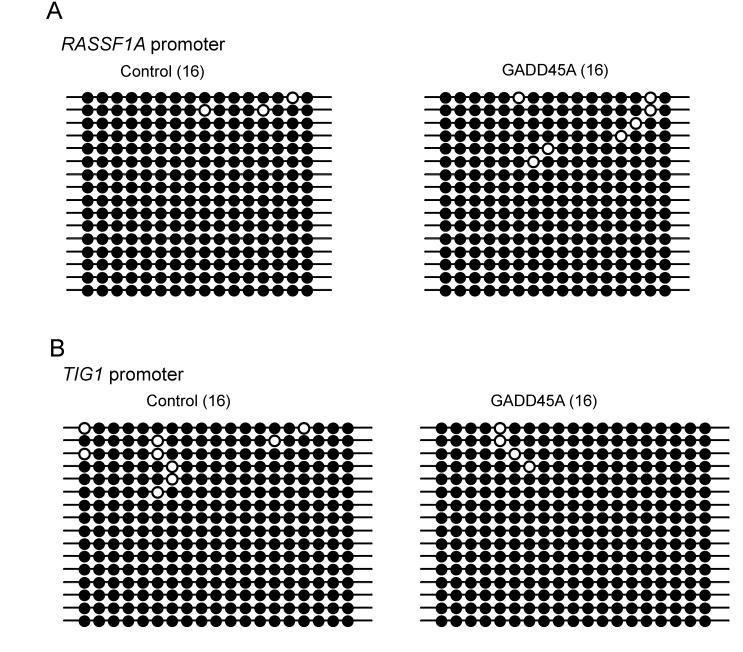
Bisulfite sequencing of two endogenous methylated genes in HEK293 cells transfected in the absence or presence of a GADD45A expression vector. The promoter sequences of the *RASSF1A* (A) and *TIG1* (B) genes are highly methylated in HEK293 cells. Transfection with the control (pcDNA3.1) vector and the *GADD45A* cDNA expression vector shows no substantial difference in genomic methylation patterns. Closed circles mark methylated CpG dinucleotides and open circles are unmethylated CpG dinucleotides.

At this point, we wished to further verify GADD45A activity towards methylation of other genomic sequences. To address this issue, we evaluated the methylation status of two endogenous single copy genes when GADD45A was overexpressed in HEK293 cells. Recently, we reported that the promoter-associated CpG islands of the *RASSF1A* and *TIG1* genes are highly methylated in HEK293 cells [30]. Therefore, we carried out bisulfite sequence analysis to see if the levels of DNA methylation at the promoters of *RASSF1A* and *TIG1* can be affected by overexpression of GADD45A in HEK293 cells. As illustrated in Figure 5, the dense methylation status of the *RASSF1A* and *TIG1* promoters generally was maintained in GADD45A-overexpressing cells.

**Figure 6:**
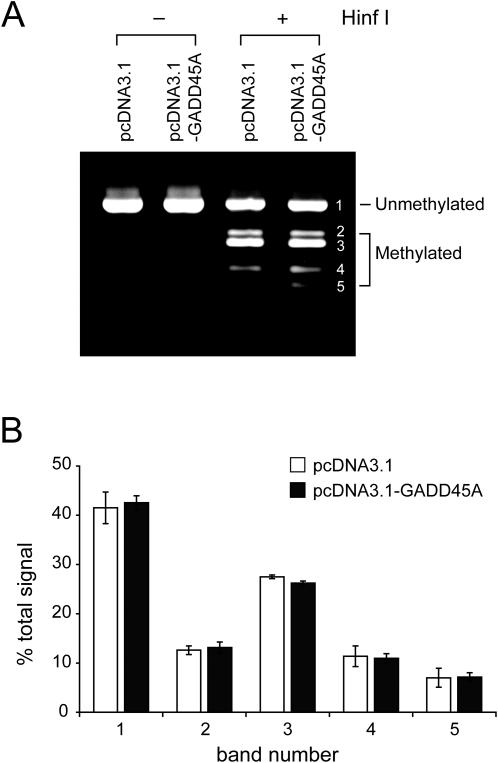
GADD45A does not promote demethylation of endogenous LINE1 sequences in human cells. HEK293 cells were transfected with pcDNA3.1 control vector or the pcDNA3.1-GADD45A expression vector. Forty-eight hours after transfection, genomic DNA was isolated and treated with sodium bisulfite. A. LINE1 promoter sequences were amplified and the PCR products were digested with HinfI, which cleaves only the methylated PCR products. B. The intensity of each cleavage product (bands 1–5) obtained from control or GADD45A-transfected cells was quantitated by densitometry. The result from three independent experiments is displayed (mean +/− S.D.).

We then analyzed methylation of endogenous LINE1 sequences in HEK293 cells. After bisulfite treatment of DNA from control cells and GADD45A overexpressing cells, we used consensus PCR primers for the LINE1 promoter. Combined bisulfite restriction analysis (COBRA) with HinfI digestion was used to assess the methylation status of these repetitive elements (Figure 6). Quantitative analysis of the cleavage products indicates that there was no difference in methylation of LINE1 sequences between control and GADD45A-transfected cells.

### Effect of co-expression of XPG

The DNA excision repair nuclease XPG contributes to repair of various types of DNA adducts. Barreto et al reported that the DNA demethylation and gene reactivation activity of GADD45A was further enhanced by co-expression of XPG or XPG in combination with XPB [1]. We used a methylated SV40-luciferase construct to test the activities of GADD45A and XPG on reporter gene activity (Figure S2). Neither the co-expression of GADD45A alone nor that of GADD45A plus XPG led to reactivation of the methylated reporter. In contrast to the previous report [1], expression of the methylated reporter gene was even further suppressed by co-expression of GADD45A.

## Discussion

It has been reported that GADD45A promotes epigenetic gene activation by active DNA demethylation presumably involving a repair-mediated process [1]. This was a surprising result since GADD45A is not expressed at the developmental stage of murine embryos at which active DNA demethylation has been well documented (Table 1). In addition, it is not immediately apparent why genomic DNA demethylation should occur under conditions where GADD45A is induced, i.e. growth arrest or DNA damage. We have not been able to confirm the biochemical results showing a role of GADD45A in DNA demethylation (Figures 1–6). The reasons for the discrepancy between our results and those of Barreto et al are not clear. We used the same cell line (HEK293) for transfection of GADD45A and the methylated reporter plasmids. The sequences analyzed for demethylation in the Oct4 promoter-EGFP construct were the same as those analyzed by Barreto et al. The methylases used and the restriction enzyme sites tested were the same. In addition to HpaII cleavage, as done by Barreto et al, we used sodium bisulfite sequencing which is considered the method of choice for high resolution DNA methylation analysis. We were also unable to confirm GADD45A-induced DNA demethylation of methylated endogenous genes (LINE1 promoter, *RASSF1A* and *TIG1*-associated CpG islands in HEK293 cells and the Oct4 promoter in NIH3T3 cells).

Barreto et al have also used GADD45A-specific siRNAs to support a role of GADD45A in DNA demethylation [1]. However, we point out that an almost 3-fold increase in global genomic methylation levels seen after knockdown of GADD45A (*see* Fig. 3c,d of their report) is virtually impossible since there are not enough unmethylated CpG dinucleotides in the human genome to allow for such an increase.

In summary, one can conclude that the identity and composition of the mammalian DNA demethylase machinery remains unknown. Viable candidate genes would be those expressed in oocytes, zygotes, and 1- or 2- cell stage embryos. AID and APOBEC1 are expressed in ovaries and oocytes [11]. Another example is the MBD3L2 protein, which is predominantly expressed at these stages (Unigene database). MBD3L2 is a homologue of the methyl-CpG binding proteins MBD2 and MBD3 that by itself does not contain a methyl-CpG binding domain [31],[32]. Work is underway in our laboratory to construct gene-targeted mice with a deletion of *Mbd3l2* in order to test the role of this gene in early mammalian development and DNA demethylation.

## Materials and Methods

### Cell culture and transfection

HEK293 and NIH3T3 cells were maintained as a monolayer in Dulbecco's modified Eagle's medium supplemented with 10% fetal bovine serum at 37°C in a 5% CO_2_ standard incubator. To serum-starve the cells, confluent HEK293 or NIH3T3 cells were cultured in a medium containing 0.5% fetal bovine serum for 48 hours after transfection. Transfection of HEK293 cells was carried out in Opti-MEM-I medium (Invitrogen; Carlsbad, CA) using Lipofectamine 2000 (Invitrogen) according to the manufacturer's instructions. Transfection of NIH3T3 cells was performed by nucleofection using the Amaxa nucleofector and kit R (Amaxa; Gaithersburg, MD), following the protocol recommended by the manufacturer. More than 50% of the NIH3T3 cells and more than 60% of the HEK293 cells were routinely transfected. For the details of the co-immunoprecipitation experiments, see Text S1.

### Western blot analysis

Total cell extracts were prepared with RIPA buffer (25 mM Tris-HCl pH 7.6, 150 mM NaCl, 1% NP-40, 1% sodium deoxycholate and 0.1% SDS) and were separated by SDS/PAGE and blotted onto PVDF membranes (BioRad). The membranes were blocked with 5% nonfat milk at 4°C overnight. After washing, GADD45A protein was detected using rabbit polyclonal IgG (Santa Cruz, sc-797) at a 1∶3,000 dilution, followed by peroxidase-conjugated anti-rabbit IgG (Santa Cruz, sc-2004) at 1∶8,000 dilution. β-tublin protein was detected using mouse monoclonal IgG (Santa Cruz, sc-5274) at 1∶400 dilution, followed by peroxidase-conjugated anti-mouse IgG (Santa Cruz, sc-2005) at 1∶5,000 dilution. The signal was visualized by using ECL-Plus (Amersham Pharmacia Biotech).

### Fluorescence microscopy

HEK293 cells were plated at a density of 2.5 × 10^5^ cells per well directly on coverslips in a six-well tissue culture dish. The transfection was achieved using Lipofectamine 2000 (Invitrogen) according to the manufacturer's instructions using 2 µg of plasmids. At 48 hours after transfection, cells were washed twice with PBS and then fixed in 3.7% formaldehyde for 5 min at room temperature. The cells were washed again, stained with 0.25 µg/ml of 4′, 6′-diamidino-2-phenylindole (DAPI) (Sigma), and mounted with 90% glycerol in PBS. Images were visualized with an Olympus IX81 fluorescence microscope.

### Plasmids and in vitro methylation

The full-length cDNA encoding human GADD45A was obtained by reverse transcription-PCR. For creating a *GADD45A* mammalian expression vector (pcDNA3.1-GADD45A), the *GADD45A* cDNA was cloned into the BamHI-XhoI sites of pcDNA3.1 (+) vector (Invitrogen). The inserted cDNA of the construct was sequenced and verified by comparing with the human *GADD45A* transcript (RefSeq; NM_001924.2) in the Ensemble database. For the pOct4-EGFP construct, the cytomegalovirus (CMV) promoter of the pEGFP-N2 vector (Clontech; Palo Alto, CA) was replaced with the 2.4-kb mouse Oct4 gene promoter. For this purpose, the AseI and EcoRI fragment of the pEGFP-N2 plasmid encoding the CMV promoter was excised by restriction nuclease cleavage. Next, the 2.4-kb Oct4 promoter region upstream of the translational start site was amplified by PCR followed by AseI and EcoRI digestion of the PCR product and insertion into the AseI and EcoRI sites of the pEGFP-N2 plasmid. The constructed pOct4-EGFP plasmid was verified by DNA sequencing. For in vitro methylation of plasmids, 10 µg each of pEGFP-N2 or pOct4-EGFP were methylated in vitro with 20 units of SssI, HpaII alone or HhaI plus HpaII DNA methylases (New England Biolabs), respectively. These methylated plasmids were then phenol/chloroform extracted, ethanol-precipitated and resuspended in TE buffer. The extent of methylation was confirmed by methylation-sensitive restriction enzyme, HpaII, digestion and evaluated with bisulfite sequencing for pOct4-EGFP (Figure 2).

### DNA methylation analysis using Southern blot

Transfected plasmids were recovered by using QIAquick PCR-purification kits (Qiagen). The plasmids were digested with HincII and HpaII and analyzed by Southern blotting using an *EGFP* gene probe.

### Bisulfite sequencing analysis

Bisulfite conversion and purification of DNA for methylation analysis was accomplished using the EpiTect Bisulfite kit (Qiagen). One µg of recovered plasmids and genomic DNAs from HEK293 or NIH3T3 cells was used for the analysis. Bisulfite modified DNA was amplified using the following primers: for analyzing the six HpaII methylation sites downstream of the Oct4 promoter of pOct4-EGFP, the forward primer 5′-TTAGAGGTTAAGGTTAGAGGGTGG-3′ and reverse primer 5′-ATAATACAAATAAACTTCAAAATCAACTTA were used. For the CpG island of the *RASSF1A* promoter, the forward primer 5′-AGTTTTTGTATTTAGGTTTTTATTG-3′ and reverse primer 5′-AACTCAATAAACTCAAACTCCCC were used. For the CpG island covering the *TIG1* promoter, the forward primer 5′-AGGAGTGGTTTTATGGGGAT-3′ and reverse primer 5′-AACCCGAACCAAAAAACAAACA-3′ [33] were used. For analyzing the methylation sites of the endogenous mouse Oct4 promoter in NIH3T3 cells, covering sequences from −1,148 to −754 upstream of the transcription start site, the forward primer 5′-GTTAGTATAGGAATGGGGGAGG-3′ and reverse primer 5′-CCATAAAACCTACACCCAAACTC-3′ were used; for sequences covering positions −289 to −24, the forward primer 5′-GGGTGTAGTGTTAATAGGTTTTGTG-3′ and reverse primer 5′-AACCAAATATCCAACCATAAAAAAA-3′ were used. The reaction buffer contained dNTPs and Hotstart Taq polymerase (Qiagen) and the samples were incubated at 95°C for 15 min and then 40 cycles of PCR at 94°C for 45 sec, 55°C for 30 sec and 72°C for 1 min, followed by a final extension step at 72°C for 5 min, were performed. The PCR products were purified using QIAquick PCR purification kits (Qiagen) and were then ligated into the pCR2.1 vector (Invitrogen). 16 to 25 colonies for each sample were sequenced. Any clones with apparent non-CpG methylation (an indication of incomplete bisulfite conversion) were excluded from the dataset, and these clones made up less than 2% of all clones sequenced.

### Global DNA methylation analysis (LINE1 PCR)

Global DNA methylation was measured by a *LINE1* methylation assay as previously reported [34]. Methylation of LINE1 elements was analyzed by bisulfite conversion of genomic DNA followed by PCR with consensus primers for the LINE1 promoter. PCR was carried out in a reaction buffer containing dNTPs and Hotstart Taq polymerase (Qiagen), and the samples were incubated at 95°C for 15 min and then 42 cycles of PCR at 94°C for 45 sec, 53°C for 1 min and 72°C for 1 min followed by a final extension step at 72°C for 7 min. The following primers were used: forward primer 5′-TTGAGTTGTGGTGGGTTTTATTTAG-3′ and reverse primer 5′-TCATCTCACTAAAAAATACCAAACA-3′. The PCR products were digested with the HinfI restriction enzyme, which cleaves only methylated DNA after bisulfite conversion. The digested PCR products were separated by electrophoresis on 2% agarose gels. The percentage of methylation was determined after imaging of the gels. The cut bands representing methylated DNA were quantitated using image analysis. The percentages represent the mean with standard deviation for triplicate samples.

## Supporting Information

Figure S1 Co-immunoprecipitation of transfected GADD45A and Aurora A- (A) Comparison of expression levels of endogenous and overexpressed GADD45A. A longer exposure of the film indicates that the level of overexpressed GADD45A is about 10-fold greater than that of the un-induced endogenous protein. The faster migrating band is most likely due to an internal ribosome entry site. (B) Co-immunoprecipitation of GADD45A and Aurora A. In order to demonstrate the biological functionality of GADD45A, we transfected HEK293 cells with pcDNA3.1-GADD45A vector together with empty Flag-vector or a Flag-Aurora-A expression plasmid (see Text S1 for details). Lysates and anti-Flag immunoprecipitates were analyzed by Western blotting using anti-GADD45A or anti-Flag antibodies.
(1.73 MB TIF)

Figure S2 Luciferase reporter assays with the methylated SV40 promoter- HEK293 cells were transfected with either unmethylated (A) or methylated (B) SV40 promoter firefly luciferase constructs. Co-transfection was done with control (pcDNA3.1) plasmid, GADD45A expression plasmid, XPG expression plasmid or a combination of both (see Text S1 for details). Firefly luciferase activity was measured and normalized to Renilla luicferase activity and expressed as F/R ratio.
(0.14 MB TIF)

Text S1 Supporting methods.
(0.05 MB DOC)
