# Supplementary figures and images for: GADD45A Does Not Promote DNA Demethylation
Source: PLoS Genet. 2008 Mar 7;4(3):e1000013. doi: 10.1371/journal.pgen.1000013 (PMC2265528; doi:10.1371/journal.pgen.1000013)

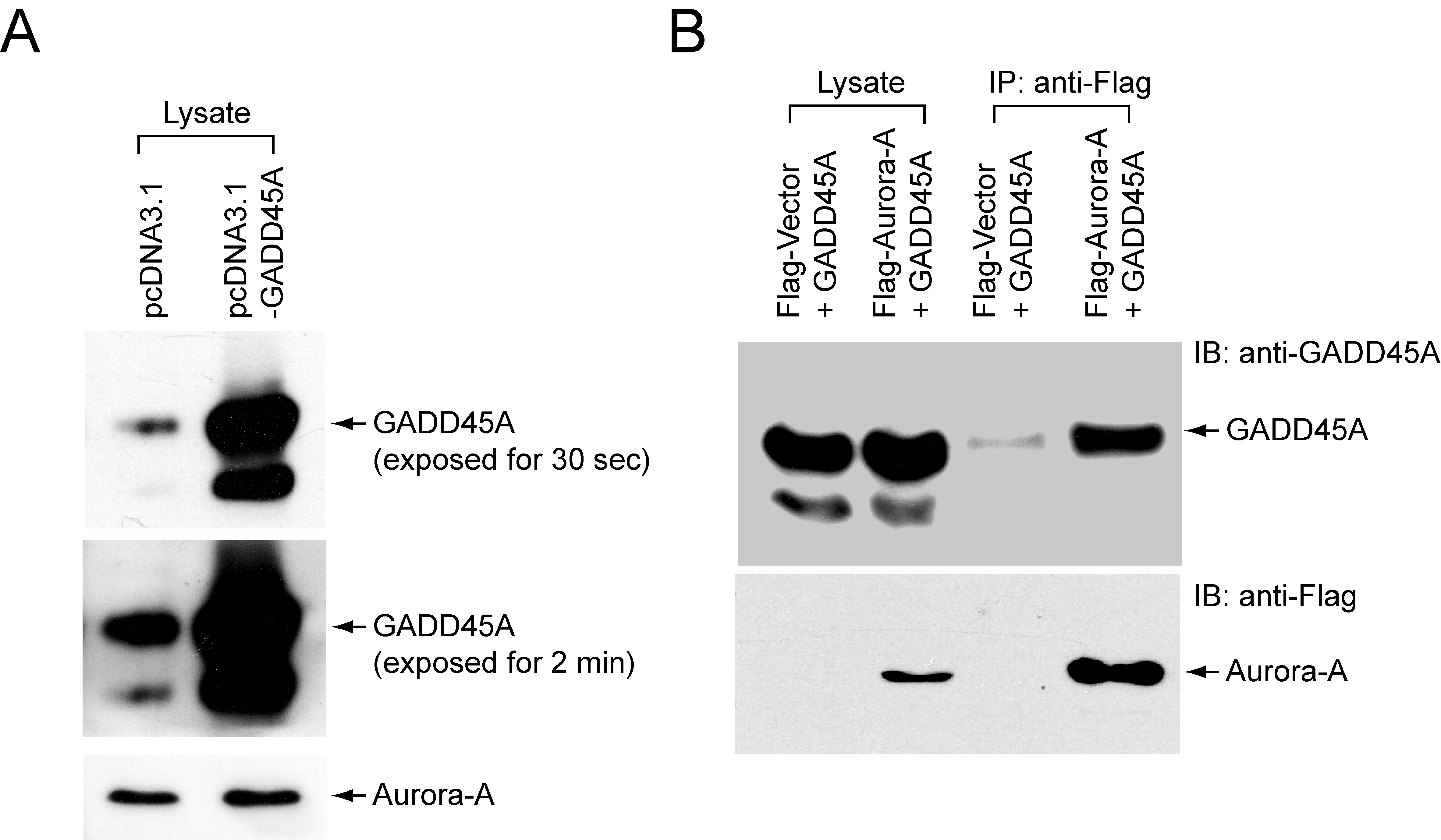

Supplement: Figure S1 — Co-immunoprecipitation of transfected GADD45A and Aurora A- (A) Comparison of expression levels of endogenous and overexpressed GADD45A. A longer exposure of the film indicates that the level of overexpressed GADD45A is about 10-fold greater than that of the un-induced endogenous protein. The faster migrating band is most likely due to an internal ribosome entry site. (B) Co-immunoprecipitation of GADD45A and Aurora A. In order to demonstrate the biological functionality of GADD45A, we transfected HEK293 cells with pcDNA3.1-GADD45A vector together with empty Flag-vector or a Flag-Aurora-A expression plasmid (see Text S1 for details). Lysates and anti-Flag immunoprecipitates were analyzed by Western blotting using anti-GADD45A or anti-Flag antibodies. (1.73 MB TIF) [file pgen.1000013.s001.tif]

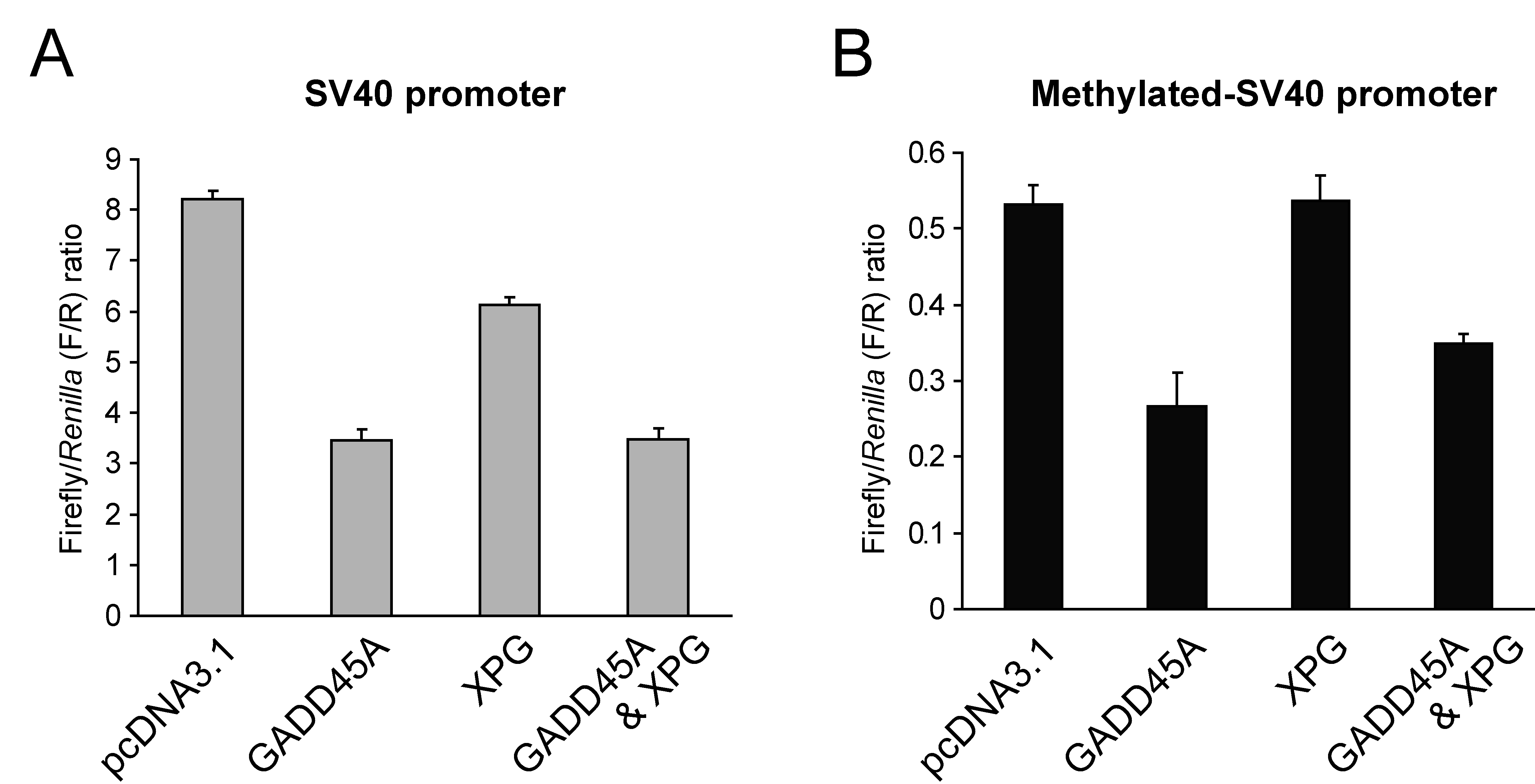

Supplement: Figure S2 — Luciferase reporter assays with the methylated SV40 promoter- HEK293 cells were transfected with either unmethylated (A) or methylated (B) SV40 promoter firefly luciferase constructs. Co-transfection was done with control (pcDNA3.1) plasmid, GADD45A expression plasmid, XPG expression plasmid or a combination of both (see Text S1 for details). Firefly luciferase activity was measured and normalized to Renilla luicferase activity and expressed as F/R ratio. (0.14 MB TIF) [file pgen.1000013.s002.tif]
