# Supplementary material for: GADD45A Does Not Promote DNA Demethylation
Source: PLoS Genet. 2008 Mar 7;4(3):e1000013. doi: 10.1371/journal.pgen.1000013 (PMC2265528; doi:10.1371/journal.pgen.1000013)
Supplement: Text S1 — Supporting methods. (0.05 MB DOC) [file pgen.1000013.s003.doc]

**Text S1**

**Jin et al.**

**GADD45A does not promote DNA demethylation**

**Supplementary Methods**

**Co-immunoprecipitation.** For immunoprecipitation, HEK293 cells were transfected with pcDNA3.1-GADD45A together with empty Flag-vector or a Flag-Aurora-A expression plasmid. Forty-eight hours after transfection, cells were harvested and lysed in TNE buffer (10 mM Tris-HCl, pH 8.0; 1% NP40; 150 mM NaCl; 1 mM EDTA) supplemented with protease inhibitor cocktail (Roche) (20 mM beta-glycerolphosphate, 50 mM sodium fluoride and 1 mM Na_3_VO_4_). After 30 min on a rotator at 4°C, lysates were collected by centrifugation at 12,200xg for 20 min at 4°C. Cell lysates were incubated with 30 µl of EZview™ Red anti-FLAG M2 affinity gel (Sigma) at 4°C on a rotator for overnight. Precipitated proteins and cell lysates were subjected to 12% SDS-PAGE followed by Western blot analysis using rabbit polyclonal anti-GADD45A IgG (Santa Cruz, sc-797) at 1:3,000 dilution and rabbit anti-Flag (Sigma), followed by peroxidase-conjugated anti-rabbit IgG (Santa Cruz, sc-2004) at 1:8,000 dilution. Aurora-A protein was detected using rabbit polyclonal IgG (Bethyl; Montgomery, TX) at 1:2,000 dilution. The signal was visualized by using ECL-Plus (Amersham Pharmacia Biotech).

**Luciferase reporter assays.** We determined the effect of co-expression of GADD45A and XPG on a methylated promoter luciferase constract. On the day before transfection, HEK293 cells were seeded in 24-well plates, then per well 150 ng of methylated or non-methylated pGL4.13 [luc2/SV40] expressing firefly luciferase reporter and 10 ng of *Renilla* luciferase reporter plasmid pGL4.74 [hRluc/TK] (Promega; Madison, WI) as an internal control reporter were cotransfected with a total 200 ng of pcDNA3.1 (for control), pcDNA3.1-GADD45A or/and pCMV-SPORTS-XPG. Methylated pGL4.13 vector was prepared by in vitro methylation with SssI DNA methylase (New England Biolabs). A human XPG expression vector for mammalian expression (pCMV-SPORTS-XPG) was kindly provided by Dr. Timothy R. O'Connor (City of Hope; Duarte, CA). Transfected HEK293 cells were cultured in medium containing 0.5% fetal bovine serum for 48 hours and then harvested. Firefly and *Renilla* luciferase activities were measured with the dual-luciferase assay (Promega; Madison, WI). Firefly activity was normalized to *Renilla* activity to control for transfection efficiency. Data represent the mean with standard deviation of triplicate samples.
